# “Food Addiction” in Patients with Eating Disorders is Associated with Negative Urgency and Difficulties to Focus on Long-Term Goals

**DOI:** 10.3389/fpsyg.2016.00061

**Published:** 2016-02-02

**Authors:** Ines Wolz, Ines Hilker, Roser Granero, Susana Jiménez-Murcia, Ashley N. Gearhardt, Carlos Dieguez, Felipe F. Casanueva, Ana B. Crujeiras, José M. Menchón, Fernando Fernández-Aranda

**Affiliations:** ^1^Department of Psychiatry, University Hospital of Bellvitge-IDIBELLBarcelona, Spain; ^2^Ciber Fisiopatologia Obesidad y Nutrición, Instituto Salud Carlos IIIBarcelona, Spain; ^3^Department of Psychobiology and Methodology, Universitat Autònoma de BarcelonaBarcelona, Spain; ^4^Department of Clinical Sciences, School of Medicine, University of BarcelonaBarcelona, Spain; ^5^Department of Psychology, University of MichiganAnn Arbor, MI, USA; ^6^Department of Physiology, Centro Singular de Investigación en Medicina Molecular y Enfermedades Crónicas, University of Santiago de Compostela-Instituto de Investigación SanitariaSantiago de Compostela, Spain; ^7^Laboratory of Molecular and Cellular Endocrinology, Research Area, Complejo Hospitalario Universitario de Santiago de CompostelaA Coruña, Spain; ^8^Ciber Salud Mental, Instituto Salud Carlos IIIBarcelona, Spain

**Keywords:** eating disorder, food addiction, personality, impulsivity, negative urgency

## Abstract

**Objectives:** The present study aimed to investigate if eating disorder patients differ in specific personality traits depending on a positive screening of food addiction (FA) and to find a model to predict FA in eating disorder patients using measures of personality and impulsivity.

**Methods**: Two hundred seventy eight patients, having an eating disorder, self-reported on FA, impulsivity, personality, eating and general psychopathology. Patients were then split into two groups, depending on a positive or negative result on the FA screening. Analysis of variance was used to compare means between the two groups. Stepwise binary logistic regression was used to obtain a predictive model for the presence of FA.

**Results:** Patients with FA had lower self-directedness, and more negative urgency and lack of perseverance than patients not reporting addictive eating. The probability of FA can be predicted by high negative urgency, high reward dependence, and low lack of premeditation.

**Conclusion:** Eating disorder patients who have more problems to pursue tasks to the end and to focus on long-term goals seem to be more likely to develop addictive eating patterns.

## Introduction

Until now there is no clear agreement about the question if FA is a valid and necessary concept, specifically in the domain of EDs. On the one hand, different components of food have been studied using animal models, providing evidence that sugar consumption – and to some extend also high fat food – can lead to addictive behaviors, similar to other substances of abuse (Teegarden and Bale, 2007; Avena et al., 2008, 2012; Gold and Avena, 2013). Hyperpalatable foods, characterized by high levels of sugar, fat and salt are potentially addictive for humans (Gearhardt et al., 2011a; Davis, 2014; Schulte et al., 2015). Apart from this, neuroimaging techniques have shed light over neural correlates of FA, as well as on the similarities between substance dependence and addictive-like eating behavior in humans in terms of reward value and incentive value of respective stimuli (Gearhardt et al., 2011b; Volkow et al., 2012; Davis et al., 2013; Smith and Robbins, 2013; Imperatori et al., 2014). On the other hand, the FA construct seems to overlap with common eating psychopathology, namely binging, and seems to have collinearity with severity of disordered eating. Furthermore, a much debated question is whether addictive properties intrinsic to specific foods (physical dependence) or rather the eating behavior *per se* (psychological dependence) play a major role in the explanation of addictive-like eating, wherefore the term “eating addiction” has been proposed in order to underline the behavioral component of these symptoms (see Hebebrand et al., 2014 for a review). This shows the need for more research on psychological processes underlying FA.

The Yale Food Addiction Scale (YFAS) was developed in 2009 with the aim to apply the diagnostic criteria for substance dependence of the fourth revision of the Diagnostic and Statistical Manual of Mental Disorders (DSM; American Psychiatric Association, 2013) to eating behavior (Gearhardt et al., 2009a). Since the development of this first validated tool for the measurement of addictive behaviors toward food, the number of publications about FA has experienced a constant growth (Gearhardt et al., 2011a). In DSM-5, the chapter on addictions has undergone reorganization, including now not only substance related disorders, but also behavioral addictions. FA could be included in this new category in future revisions of the DSM.

A meta-analysis including 23 studies using the YFAS reports a mean prevalence of FA of 19.9% in adult samples ranging from healthy normal weight, over obesity, to BED, and BN, in which the highest prevalence of up to 100% was found (Pursey et al., 2014). In a recent study using the YFAS in ED patients, 72.8% of the sample fulfilled the criteria for FA compared to 2.4% of healthy controls, those ED patients who report FA showing higher ED severity and more general psychopathology (Granero et al., 2014). If ED patients with and without FA differ on basic psychological measures, such as personality and impulsivity traits, focused approaches to treatment may be helpful. However, there is a lack of literature analyzing personality vulnerabilities underlying FA.

The idea, that personality characteristics implicated in addictive processes could also contribute to ED, is not a new concept and has been confirmed by empiric data (Davis and Claridge, 1998; Lent and Swencionis, 2012). ED patients are more likely than healthy controls to use addictive substances such as tobacco, but also illicit drugs (Krug et al., 2008), which supports the notion of an “addictive personality.” Yet, it is possible that this association is explained by those patients fulfilling the criteria of FA, rather than being typical to all ED patients. Assuming that FA is comparable to other (substance and/or behavioral) addictions, it is expectable that, after controlling for ED subtypes, patients having a positive FA screening will have more addictive-like personality traits than those who do not fulfill the YFAS criteria for FA.

A recent meta-analysis on temperament in ED (Atiye et al., 2015) shows high harm avoidance in all ED-types compared to controls, high novelty seeking in BN patients, high persistence in AN, BN and Other Not Specified Eating or Feeding Disorders (OSFED), and no differences in reward dependence between patient and control groups. Furthermore, all types of ED-patients were found to have lower scores in self-directedness than healthy controls (Fassino et al., 2004). By comparison, the personality profile found in individuals with substance related and non-substance related addictive disorders, namely gambling disorder, shows similarities but also differences: high novelty seeking and low self-directedness was reported transdiagnostically for different drugs (Le Bon et al., 2004; Pedrero Pérez and Rojo Mota, 2008) and non-substance related addictions (Alvarez-Moya et al., 2007), harm avoidance in contrast may vary depending on the substance consumed (Schneider et al., 2015) and on sex (Clinton et al., 2004; Claes et al., 2012a; Granero et al., 2014). When comparing behavioral addictions (gambling disorder, compulsive buying) to BN, high novelty seeking is more specifically related to the former group, whereas low self-directedness is associated to both groups and reward dependence is not clearly related to either of the groups (Alvarez-Moya et al., 2007; Jiménez-Murcia et al., 2015). Harm avoidance in general is high in both clinical groups, but may be a more gender specific trait, with lower values in males than in females (Alvarez-Moya et al., 2007; Claes et al., 2012a).

Since impulsivity is an important characteristic common to behavioral and substance addictions (Lawrence et al., 2009; Alvarez-Moya et al., 2011; Kaiser et al., 2012; Jiménez-Murcia et al., 2013; Ochoa et al., 2013; Torres et al., 2013; Di Nicola et al., 2015), heightened levels could also be associated with FA. However, high impulsivity has also been found in ED patients (Davies et al., 2009; Claes et al., 2012b, 2015), wherefore a clarification is needed of whether this correlate is related to ED in general, or if it relates specifically to addictive-like eating. In studies using different self-report measures (UPPS, Barratt Impulsivity Scale) in student populations, high impulsivity was related to higher scores on the YFAS (Davis et al., 2011); more specifically, negative urgency, lack of perseverance (Murphy et al., 2014; Pivarunas and Conner, 2015) and attentional impulsivity (Meule et al., 2012; Raymond and Lovell, 2015), while motor and non-planning impulsivity were related to FA only in one (Raymond and Lovell, 2015) of these studies. Regarding behavioral response inhibition tasks, FA was not consistently related to task performance (Meule et al., 2012, 2014a). These results show that the term “impulsivity” has been referred to in different ways and with varying meanings, which may explain the discrepant results of self-report measures of impulsivity when compared to behavioral impulsivity tasks (Cyders and Coskunpinar, 2011; Meule et al., 2014a) and shows that a clear definition of this construct is needed. In the following, impulsivity will be defined according to a five factor-model (Cyders et al., 2007) incorporating the facets lack of premeditation, lack of perseverance, sensation seeking, positive urgency and negative urgency.

The objectives of the present study were (1) to investigate if ED patients differ in specific personality traits depending on a positive FA screening according to the YFAS; and (2) to find a model to predict FA in ED patients using measures of personality and impulsivity. More specifically, starting from the literature on addictive personality traits, it was hypothesized that ED patients with FA would have more novelty seeking, similar self-directedness, reward dependence and harm-avoidance (1a), and higher negative urgency and lower perseverance than ED patients without FA (1b). The second objective was more explorative; therefore, we did not make specific hypotheses on which variables would best predict FA.

## Materials and Methods

### Participants

Participants (*n* = 278, 20 males) were recruited from consecutive referrals to the ED Unit of the Department for Psychiatry of Bellvitge University Hospital during a period comprised from September 2013 until March 2015. AN (*n* = 68), BN (*n* = 110), BED (*n* = 39), and OSFED (*n* = 61) patients were originally diagnosed according to DSM-IV-TR (American Psychiatric Association, 2000) criteria by means of the Structured Clinical Interview for DSM Disorders-I (First et al., 1996), conducted by experienced psychologists and psychiatrists. DSM-IV diagnoses were reanalyzed *post hoc* using the recent DSM-5 criteria to ensure diagnoses reflected the current diagnostic criteria (American Psychiatric Association, 2013). See **Table 1** for sociodemographic variables, for further information on the sample characteristics see Supplementary Tables S1 and S2.

**Table 1:** Demographic and selected clinical data for the sample.

	Total *n* = 278		AN *n* = 68		BN *n* = 110		OSFED *n* = 61		BED *n* = 39		*χ^2^*	*df*	*p*
Gender females	92.8%		89.7%		96.4%		95.1%		84.6%		7.46	3	0.059
Employed	69.4%		58.8%		70.0%		78.7%		71.8%		6.19	3	0.103
Tobacco use	30.6%		29.4%		31.8%		39.3%		15.4%		6.57	3	0.087
Alcohol abuse	10.1%		7.4%		17.3%		6.6%		0%		12.1	3	0.007
Other drugs use	12.9%		10.3%		19.1%		9.8%		5.1%		6.75	3	0.080
Food addiction: screening positive	74.8%		55.9%		89.1%		62.3%		87.2%		33.08	3	<0.001

	**Mean**	***SD***	**Mean**	***SD***	**Mean**	***SD***	**Mean**	***SD***	**Mean**	***SD***	***F***	***df***	***p***

Age (years-old)	29.1	10.4	26.7	9.2	28.7	9.4	26.6	10.2	38.3	10.9	14.4	3; 274	<0.001
Age of onset (years-old)	19.7	8.4	17.7	5.1	19.0	7.0	19.8	9.9	24.6	11.8	5.8	3; 274	0.001
Duration of illness (years)	9.3	9.0	8.3	9.1	9.8	8.7	6.9	6.9	13.6	11.1	4.6	3; 274	0.004
BMI (kg/m^2^)	24.5	8.7	16.8	1.5	25.2	6.4	22.7	4.2	38.9	9.1	125.0	3; 274	<0.001
Food addiction: total criteria	4.76	1.89	3.51	1.71	5.69	1.46	3.74	1.72	5.92	1.36	42.93	3; 274	<0.001

*AN, anorexia nervosa; BED, binge eating disorder; BMI, Body Mass Index (kg/m^2^); BN, bulimia nervosa; OSFED, Other Not Specified Eating or Feeding Disorders; SD, standard deviation.*

### Assessment

#### Yale Food Addiction Scale-Spanish Version -YFAS-S (Gearhardt et al., 2009b; Granero et al., 2014)

The YFAS measures FA using 25 items which are assigned to seven scales, referring to the seven criteria for substance dependence defined by the DSM-IV: (1) tolerance, (2) withdrawal, (3) substance taken in larger amount/period of time than intended, (4) persistent desire/unsuccessful efforts to cut down, (5) great deal of time spent to obtain substance, (6) important activities given up to obtain substance, (7) use continued despite psychological/physical problems (American Psychiatric Association, 2000). The YFAS was translated into Spanish and validated in the Spanish adult and ED population, with good validity and reliability scores (Granero et al., 2014).

For the following analyses, we either used the “FA total criteria,” which indicates the number of fulfilled subscales, or the positive versus negative screening result. If at least three of the seven criteria are met for a period of the last 12 months and the person feels significantly impaired and/or suffers due to the described behavior, this is referred to as “positive YFAS screening score.” Internal consistency for the YFAS in our sample was excellent, Cronbach’s α = 0.92.

#### UPPS-P Impulsive Behavior Scale-UPPS (Whiteside and Lynam, 2001; Cyders et al., 2007)

The UPPS-P measures five facets of impulsive behavior through self-report on 59 items: positive and negative urgency (tendency to act rashly in response to positive mood or to distress), lack of perseverance (inability to remain focused on a task), lack of premeditation (tendency to act without thinking of the consequences of an act) and sensation seeking (tendency to seek out novel and thrilling experiences). The Spanish translation shows good reliability (Cronbach’s α between 0.79 and 0.93) and external validity (Verdejo-García et al., 2010). Reliability as measured by Cronbach’s α for the UPPS-P in the study sample ranged from very good (negative urgency α = 0.83) to excellent (positive urgency α = 0.91).

#### Temperament and Character Inventory-Revised-TCI-R (Cloninger, 1994)

The TCI-R is a 240-item self-report questionnaire measuring personality on four temperament and three character dimensions. The temperament dimensions are harm avoidance (inhibited, passive vs. energetic, outgoing); novelty seeking (approach to signals of reward, impulsivity vs. uninquiring, reflective); reward dependence (sociable, socially dependent vs. tough-minded, socially insensitive) and persistence (perseverant, ambitious vs. inactive, erratic). Character covers self-directedness (responsible, goal-directed vs. insecure, inept); cooperativeness (helpful, empathic vs. hostile, aggressive) and self-transcendence (imaginative, unconventional vs. controlling, materialistic). The original questionnaire and the Spanish version of the revised questionnaire were validated and show good psychometric properties (Cloninger, 1994; Gutiérrez-Zotes et al., 2004). Internal consistency for the TCI-R in the study sample ranged from very good (novelty seeking α = 0.80) to excellent (harm avoidance α = 0.91).

#### Eating Disorders Inventory-2-EDI-2 (Garner et al., 1983)

The EDI-2 is a 91-item self-report questionnaire that assesses characteristics of AN and BN on the dimensions drive for thinness, bulimia, body dissatisfaction, ineffectiveness, perfectionism, interpersonal distrust, interoceptive awareness, maturity fears, asceticism, impulse regulation and social insecurity. This scale has been validated in a Spanish population (Garner, 1998), obtaining a mean internal consistency of α = 0.63.

#### Symptom Check-List 90-Revised-SCL-90-R (Derogatis, 1994)

The SCL-90-R is a self-report questionnaire measuring psychological distress and psychopathology through 90 items. The items load on nine symptom dimensions: somatization, obsessive–compulsive, interpersonal sensitivity, depression, anxiety, hostility, phobic anxiety, paranoid ideation and psychoticism. The global score (Global Severity Index, GSI), is a widely used index of psychopathological distress. The SCL has been validated in a Spanish sample obtaining a mean internal consistency of α = 0.75 (Derogatis, 2002).

#### Behavioral and Substance Addictions

Gambling, kleptomania, stealing and buying behavior and the abuse of alcohol, the use of tobacco (smoking on an at least a daily basis) and drugs (lifetime use of any drug other than alcohol and tobacco) were assessed in a clinical interview conducted by psychologists and psychiatrists experienced in the field of addictive behaviors.

### Procedure

This study was approved by the local ethics committee and was conducted according to the Declaration of Helsinki. After participants signed informed consent, they were evaluated and diagnosed at the ED Unit of the University Hospital of Bellvitge by experienced psychologists and psychiatrists, who conducted two semi-structured face-to-face interviews. The first interview provided information about current ED symptoms, antecedents and other psychopathological data of interest. The second interview comprised psychometrical assessment, and weight (assessment of body mass index and body composition) and eating monitoring (through daily reports completed at home on food intake, purges, and binges).

### Statistical Data Analyses

Statistical analyses were conducted with SPSS20 for windows. Since age significantly differed between groups and ED subtype is known to influence the probability of FA (Granero et al., 2014), these two variables were entered as covariates. ANOVA, adjusted by participants’ age and ED subtype, was used to compare the means of the seven TCI-R and the five UPPS-P subscales between participants classified into the two FA groups (positive and negative screening score).

Regarding missing data, statistical analyses were performed for subjects with complete information on each instrument (pair-wise procedure). The number of missing data was very low in this study: only data from one SCL-90R questionnaire were missing (for one patient in the YFAS-negative group), one TCI-R (also for one patient in the YFAS-negative group) and eight UPPS (two patients of YFAS-negative and six patients of YFAS-positive group).

Stepwise binary logistic regression was used to obtain a predictive model for the outcome presence of a “positive YFAS screening score” (more than three criteria fulfilled and clinical significance), considering three blocks: the first block included and fixed the participants’ sex, age and diagnostic subtype, the second block automatically selected the TCI-R scales with a significant prediction on the dependent variable, and the third block selected the UPPS-P scales with significant contribution. The predictive capacity of each block was measured through the increase in the Nagelkerke’s pseudo-*R*^2^ coefficient and the goodness-of-fit of the final model through the Hosmer and Lemeshow test (Hosmer et al., 2013). Due to the multiple statistical comparisons, Bonferroni-Finner correction was included to avoid the increase in Type-I errors. The measure of the effect size for mean and proportion comparisons was done through the 95% confidence interval of the parameters and the Cohen’s-*d* coefficient (moderate effect size was considered for |*d*| > 0.50 and high effect size for |*d*| > 0.80).

## Results

### Temperament, Character and Impulsivity Traits in ED Patients with and without Food Addiction

**Table 2** shows the results of the ANOVA comparing the temperament and character (TCI-R) and impulsivity (UPPS-P) traits mean scores between patients with positive versus negative YFAS screening score, adjusted by age and ED subtype. The analysis was carried out in two steps. In the first step the interaction parameter “positive YFAS screening score” by ED-subtype was included into the ANOVA to assess whether differences between individuals with positive and negative YFAS screening score were related to the different ED subtypes. Since this interaction term was not statistically significant, it was excluded from the model and the main effects of a “positive YFAS screening score” were estimated and interpreted. Results show that ED patients with positive FA screening compared to patients without FA have lower self-directedness (*p* < 0.01), while novelty seeking (*p* = 0.915), harm-avoidance (*p* = 0.08) and reward dependence (*p* = 0.56) do not differ significantly between groups. For a graphical representation and norm comparisons, see Supplementary Figure S1.

**Table 2:** Differences on mean scores of personality traits and impulsivity for patients with or without food addiction: ANOVA adjusted by age and ED subtype.

	Adjusted means; *SD*				ANOVA						
	FA = negative		FA = positive		FA × ED		(adjusted by age and ED subtype)				
	*n* = 70		*n* = 208		*F_df__=__3;275_*	*^1^p*	*F_df__=__1;275_*	*^1^p*	*eta^2^*	MD	|*d*|
TCI-R: Novelty seeking	100.39	15.07	100.71	15.83	0.36	0.781	0.02	.915	0.000	0.32	0.02
TCI-R: Harm avoidance	112.89	19.54	119.91	21.08	1.33	0.266	5.24	.080	0.019	7.02	0.35
TCI-R: Reward dependence	99.40	16.89	101.78	15.62	0.23	0.876	0.99	0.562	0.004	2.38	0.15
TCI-R: Persistence	105.90	18.37	106.24	22.68	0.42	0.739	0.01	0.915	0.000	0.34	0.02
TCI-R: Self-directedness	125.32	21.63	115.37	20.46	0.59	0.622	11.17	**0.007**	0.040	-9.95	0.47
TCI-R: Cooperativeness	136.49	17.33	134.07	16.24	0.29	0.835	1.02	0.562	0.004	-2.43	0.14
TCI-R: Self-Transcendence	63.32	13.28	63.88	14.27	2.00	0.114	0.07	0.915	0.000	0.57	0.04

UPPS: Lack premeditation	23.48	6.08	23.38	6.24	0.07	0.974	0.01	0.912	0.000	-0.10	0.02
UPPS: Lack perseverance	21.44	5.45	23.54	5.96	0.79	0.500	6.22	**0.033**	0.023	2.10	0.37
UPPS: Sensation seeking	27.50	8.01	25.27	8.80	0.71	0.546	3.41	0.110	0.013	-2.23	0.26
UPPS: Positive UR	27.07	8.79	29.13	8.99	0.21	0.892	2.34	0.159	0.009	2.05	0.23
UPPS: Negative UR	29.68	6.70	34.39	6.56	0.22	0.881	24.50	**<0.001**	0.085	4.70	**0.71**^∗^

*FA, food addiction; ED, eating disorder; FA × ED, interaction parameter; MD, mean difference. eta^2^, partial eta^2^. ^1^p: includes Bonferroni-Finner correction for multiple statistical comparisons. Bold: significant comparison (0.05 level). ^∗^Bold: moderate (|d| > 0.50) to high ((|d| > 0.80) effect size.*

There were significant differences on the UPPS-P subscales lack of perseverance (*p* < 0.05) and negative urgency (*p* < 0.001), with higher values in FA patients compared to patients without “positive YFAS screening score” (see **Table 2**). Lack of premeditation, sensation seeking and positive urgency did not differ as a function of FA.

### Predictive Capacity of Personality in the Explanation of Food Addiction

**Table 3** includes the final predictive model of the presence of a positive YFAS screening score. The first block, including the covariates sex, age, and diagnostic subtype, obtained an initial predictive capacity equal to *R^2^* = 0.22. In the second block, the TCI-R reward-dependence and self-directedness scale scores were selected and fixed, with an increase in the predictive capacity equal to *R^2^* = 0.08, while the other TCI-R traits didn’t explain further variance. In the third block, the UPPS-P lack of premeditation and negative urgency scores were included, and the new increase in the predictive ability was *R^2^* = 0.08, while the other UPPS-P subscales did not add additional explanative power. The final predictive model contained in the third block of the logistic regression indicates that after adjusting for sex, age, and ED subtype, the odds of a “positive YFAS screening score” is increased by high scores in the reward-dependence and negative urgency scales and low scores in the lack of premeditation scale, while negative urgency can be seen as the strongest predictor of FA. This model achieved goodness-of-fit (Hosmer–Lemeshow test: *p* = 0.408).

**Table 3:** Predictive model for the dependent variable: positive screening of food addiction.

Criterion: FA “positive YFAS screening score”		*B*	*SE*	Wald	*p*	OR	95%CI (OR)	
**First block (Δ*R*^2^ = 0.221)**								
Sex (0 = female, 1 = male)		-0.369	0.596	0.383	0.536	0.69	0.22	2.22
Age (years-old)		0.001	0.018	0.006	0.938	1.00	0.97	1.04
Diagnostic subtype	*AN* vs. *OSFED*	-0.293	0.398	0.541	0.462	0.75	0.34	1.63
	*BN vs. OSFED*	1.674	0.450	13.829	<0.001	5.33	2.21	12.88
	*BED vs. OSFED*	2.258	0.813	7.707	0.006	9.57	1.94	47.11
***Second block (Δ*R*^2^ = 0.078)***								
Sex (0 = female, 1 = male)		-0.039	0.658	0.004	0.953	0.96	0.26	3.49
Age (years-old)		0.001	0.019	0.002	0.964	1.00	0.97	1.04
Diagnostic subtype	*AN* vs. *OSFED*	-0.011	0.420	0.001	0.978	0.99	0.43	2.25
	*BN* vs. *OSFED*	1.644	0.470	12.239	<0.001	5.18	2.06	13.00
	*BED* vs. *OSFED*	2.064	0.828	6.208	0.013	7.88	1.55	39.95
TCI-R: Reward-dependence		0.023	0.011	3.897	0.048	1.02	1.00	1.05
TCI-R: Self-directedness		-0.030	0.009	12.154	<0.001	0.97	0.95	0.99
***Third block (Δ*R*^2^ = 0.079)***								
Sex (0 = female, 1 = male)		0.095	0.709	0.018	0.894	1.10	0.27	4.41
Age (years-old)		0.000	0.020	0.000	0.999	1.00	0.96	1.04
Diagnostic subtype	*AN* vs. *OSFED*	-0.048	0.452	0.011	0.916	0.95	0.39	2.31
	*BN* vs. *OSFED*	1.517	0.486	9.757	0.002	4.56	1.76	11.81
	*BED* vs. *OSFED*	2.004	0.843	5.658	0.017	7.42	1.42	38.70
TCI-R: Reward-dependence		0.026	0.012	4.531	0.033	1.03	1.00	1.05
TCI-R: Self-directedness		-0.016	0.011	2.209	0.137	0.98	0.96	1.01
UPPS: Lack premeditation		-0.069	0.033	4.235	0.040	0.93	0.87	1.00
UPPS: Negative UR		0.124	0.034	12.992	<0.001	1.13	1.06	1.21
Constant		-2.439	2.358	1.070	0.301	0.09		

*OSFED, Other Not Specified Eating or Feeding Disorders; AN, anorexia nervosa; BN, bulimia nervosa; BED, binge eating disorder; SE, Standard Error; OR, Odds Ratio; CI, Confidence Interval.*

## Discussion

Our first goal was to determine if ED patients with FA differ in personality traits when compared with ED Patients without FA, after controlling for ED subtypes and age. Prevalence of FA is high in ED (Gearhardt et al., 2013; Granero et al., 2014; Meule et al., 2014b), in our sample 74.8% of participants met criteria for FA. Those with comorbid FA indeed showed a distinct personality profile, although it was different than expected from the literature regarding “addictive personality traits.” FA was not related to higher values in novelty seeking, but exclusively to lower self-directedness (1a). With regard to impulsivity, the hypothesis that ED patients with FA would have higher lack of perseverance and lower negative urgency was supported by our data (1b).

Lower self-directedness has been found to be a characteristic trait both in individuals with substance related and non-substance related addictive disorders, and seems to identify individuals more vulnerable to develop addictive behavior patterns (Alvarez-Moya et al., 2007; Schneider et al., 2015). In ED patients, low self-directedness is also a characteristic trait (Fassino et al., 2002; Cassin and Von Ranson, 2005; Alvarez-Moya et al., 2007), but those with FA seem to be even more marked in this regard. Further support for our results is provided by another study (Bégin et al., 2012), that examined personality differences between overweight/obese women with and without FA and found that women with FA were more similar to women with substance use disorder than women without FA, particularly in regard to impulsivity and self-directedness.

Research has shown that harm avoidance is common to all ED subtypes and significantly higher in patients compared to controls (Cassin and Von Ranson, 2005; Lilenfeld et al., 2006; Atiye et al., 2015). In our study, both ED groups had values beyond the norms of general population (see Supplementary Figure S1), but no significant association was found between this temperament factor and a higher rate of FA. According to this data, we can thus infer that patients high in FA seem to have more problems with goal-orientation and accountability (as measured by self-directedness) compared to ED patients without FA, but both groups are comparable in behavioral and social inhibition and fear of uncertainty (as measured by harm-avoidance). Low self-directedness in patients high in FA implicates that this group has poor resourcefulness; this may present itself in problems to realistically adapt behavior to environmental requirements and to remain in accord with individual goals at the same time. Patients low in self-directedness may also be blaming and unreliable, which could lead to interpersonal problems in this patient group.

The results of this study further indicate that patients reporting addictive eating patterns have more difficulties to pursue tasks to the end and to focus on long-term goals, especially when they are in a negative mood. This is reflected by their high lack of perseverance and high values of negative urgency and is consistent with the results reported for non-clinical populations (Murphy et al., 2014; Pivarunas and Conner, 2015). It is interesting to note that FA patients show high impulsivity related to the regulation of *negative* emotions (as measured by negative urgency), but do not show elevated values in impulsivity related to *positive* emotions (as measured by positive urgency). Negative emotions may signal a discrepancy between personal needs and present conditions, which for individuals with high negative urgency is hard to bear (Cyders and Smith, 2008). This suggests that patients with FA feel a strong pressure to act immediately when having negative emotions instead of enduring until a moment more suitable to change. Since the need by itself all too often cannot be fulfilled immediately, ingestion of rewarding food can be seen as an attempt to escape these unbearable emotions by other means, which – depending on subjective expectancies – could also be a drug or another behavior (Fischer et al., 2012; Torres et al., 2013). Previous research shows that FA is also related to difficulties in emotion regulation (Gearhardt et al., 2012; Pivarunas and Conner, 2015), which corroborates the results on impulsive acts related to negative mood states.

Unexpectedly, ED patients with FA did not show elevated levels of novelty seeking when compared to ED patients without FA. In general, therefore, it seems that the approach to appetitive stimuli (reward seeking), which is implied by novelty/sensation seeking, does not differ between ED patients with and without addictive eating behavior. This points out that FA as assessed by the YFAS is more related to negative rather than to positive reinforcement, which is in line with results of a former study in normal weight participants (Meule and Kübler, 2012). It has been proposed that sensation seeking may be related rather to non-clinical drug use, than to an actual addiction (Torres et al., 2013), which would explain why patients with FA do not necessarily show elevated levels of sensation/novelty seeking.

In regard to the study’s second objective, higher values in reward dependence, negative urgency and lack of premeditation and lower values in self-directedness together explained about 15% of the variance on having or not a positive FA screening, over and above sex, age, and diagnostic subtype, while negative urgency was the most important predictor and reduced the predictive power of the other variables to very small effects. Until now, risk factors for suffering FA have been established in different samples, e.g., students (Murphy et al., 2014; Pivarunas and Conner, 2015), obese women with overeating problems (Bégin et al., 2012) or in ED patients (Gearhardt et al., 2013; Granero et al., 2014; Meule et al., 2014b), but no study has explored which would be the highest risk population for presenting FA. Our prediction model suggests that individuals with a high disposition to act rashly to negative emotions are highly vulnerable for FA and would benefit from a specific approach for treating FA symptoms.

It is important to bear in mind the cross-sectional nature of our study; we cannot definitely conclude if the personality traits found to be related to FA precede or succeed FA symptoms, or if both have one common cause. Further work is required to confirm the interrelations between different predictors of FA in ED patients. Another limitation of this study is the small sample size, especially for male patients, wherefore results on effects of gender in FA should be investigated in future studies with higher sample power. Furthermore, our study only included one self-report measure of FA, which could be completed by measures of craving, daily assessments and behavioral food ingestion tests in future studies.

Regarding the YFAS, a key issue is the high prevalence rates of FA in AN patients, which seems counterintuitive. Nevertheless, looking at the “total criteria fulfilled” (see **Table 1**), it appears that AN patients have a smaller number of total criteria fulfilled compared to BN and BED; this may indicate to some part a problem of the cut-off criteria of the YFAS. In addition to this, our results show that the criteria most frequently fulfilled in AN patients are “important activities given up” (60.3%) and “unable to cut down/stop” (89.7%) (see Supplementary Table S3). Some of the items of the YFAS, such as those loading on “important activities given up” and “impairment or distress” may apply to AN in a similar way as to patients on the bulimic spectrum, wherefore this patient group also scores high on these criteria. On the other hand, the subscale “unable to cut down or stop” seems to be systematically misunderstood by AN patients, possibly due to their subjective feeling of eating too much. This could be addressed in future revisions of the scale and should be born in mind when employing the YFAS in this patient group.

It has been formerly suggested that FA may merely be an index of ED severity (Davis, 2013; Gearhardt et al., 2014). The data at hand suggests that ED patients with FA apart from showing a more severe symptomatology may differ from those without FA in the reward value they expect from food intake. Rather than enjoying the hedonic value of food in good mood, ED patients scoring high on FA mainly use food to regulate their negative emotions. It can be hypothesized that the relation between negative emotional states and food intake is mediated by impulsive personality traits and problems to focus on basic values or personal goals.

To improve the described emotional dysregulation and inhibition of responses, a training of emotion regulation strategies such as acceptance of emotional states could be helpful (Murakami et al., 2015). The importance to integrate work on emotions and emotion regulation skills into cognitive behavioral psychotherapy has reached increasing recognition in the last years (Kahl et al., 2012; Moyal et al., 2015), and new therapy approaches for ED patients have been developed. One example is the Cognitive Remediation and Emotion Skills Training (CREST), a manualized brief psychotherapy addressing emotion regulation and recognition (Money et al., 2011; Tchanturia et al., 2015), where patients learn to differentiate between different emotions and are taught about the communicative function of negative emotions. Patients with addictive-like eating patterns might benefit from this kind of training; the findings of our study further suggest that work on value-oriented behavior is important for patients with FA. Furthermore, this patient group might benefit to a great extend from learning to endure negative emotions by the use of strategies other than food intake and by this means they may be able to gradually reduce their dependence on food/eating in order to regulate negative mood states.

The psychological basis of addictive-like eating compared to mere ED, e.g., the importance ascribed to body shape, food-related cognitions, emotion regulation, should be further investigated in future studies. Which situations and emotional states lead to uncontrolled food intake in each group and the cognitions going along with this behavior could be investigated in experimental studies or ecological momentary assessment studies.

## Author Contributions

IW and IH contributed to the design of the work, acquisition and interpretation of the data. RG was responsible for statistical analysis and for writing statistical sections of the manuscript. SJ-M, AG contributed to administering and interpreting the psychological tests of this study. CD, FC, AC, JM, FF-A participated in the design of the study. All authors (IW, IH, RG, SJ-M, AG, CD, FC, AC, JM, FF-A) contributed to critically revising the work, approved the final version of the article to be published and agreed to be accountable for all aspects of the work in ensuring that questions related to the accuracy or integrity of any part of the work are appropriately investigated and resolved.

## Conflict of Interest Statement

The authors declare that the researchwas conducted in the absence of any commercial or financial relationships that could be construed as a potential conflict of interest. The reviewer Özgür Albayrak and handling Editor Astrid Müller declared their shared affiliation, and the handling Editor states that the process nevertheless met the standards of a fair and objective review.

## Abbreviations

AN: anorexia nervosa
ANOVA: analysis of variance
BED: binge eating disorder
BN: bulimia nervosa
DSM: Diagnostic and Statistical Manual of Mental Disorders
ED: eating disorder
FA: food addiction
OSFED: other specified feeding or eating disorders
TCI: temperament and character inventory
YFAS: Yale Food Addiction Scale
