# Supplementary material for: “Food Addiction” in Patients with Eating Disorders is Associated with Negative Urgency and Difficulties to Focus on Long-Term Goals
Source: Front Psychol. 2016 Feb 2;7:61. doi: 10.3389/fpsyg.2016.00061 (PMC4735728; doi:10.3389/fpsyg.2016.00061)
Supplement: Supplementary file 1 [file Data_Sheet_1.DOCX]

**Supplementary Material**

**Table S1**: Differences in ED severity and general psychopathology for patients with negative versus positive screening for food addiction: ANOVA adjusted by age and ED subtype.

|  | Adjusted means; SD | | | | ANOVA | | | |  |
| --- | --- | --- | --- | --- | --- | --- | --- | --- | --- |
|  | FA=negative | | FA=positive | | (adjusted by age and ED subtype) | | | |  |
|  | *n*=70 | | *n*=208 | | *F_df=1;275_* | *^1^p* | *eta^2^* | MD | \|*d*\| |
| EDI-2: Drive for thinness | 10.43 | 6.88 | 14.46 | 5.14 | 27.96 | **<.001** | .093 | 4.02 | **0.66*** |
| EDI-2: Body dissatisfaction | 14.14 | 8.18 | 18.17 | 7.62 | 13.86 | **<.001** | .048 | 4.03 | **0.51*** |
| EDI-2: Interoceptive awar. | 7.48 | 5.76 | 12.37 | 6.66 | 27.50 | **<.001** | .092 | 4.90 | **0.79*** |
| EDI-2: Bulimia | 4.37 | 3.68 | 7.57 | 5.30 | 29.14 | **<.001** | .097 | 3.20 | **0.70*** |
| EDI-2: Interpers. distrust | 5.93 | 4.64 | 6.14 | 5.02 | 0.08 | .777 | .000 | 0.21 | 0.04 |
| EDI-2: Ineffectiveness | 8.96 | 6.60 | 12.04 | 7.87 | 7.74 | **.009** | .028 | 3.08 | 0.42 |
| EDI-2: Maturity fears | 8.78 | 5.87 | 8.34 | 5.82 | 0.26 | .664 | .001 | -0.44 | 0.08 |
| EDI-2: Perfectionism | 4.49 | 3.63 | 5.86 | 4.43 | 4.80 | **.039** | .017 | 1.37 | 0.34 |
| EDI-2: Impulse regulation | 4.36 | 4.94 | 7.18 | 6.21 | 10.72 | **.002** | .038 | 2.82 | **0.50*** |
| EDI-2: Ascetism | 4.90 | 3.84 | 7.60 | 4.17 | 20.29 | **<.001** | .069 | 2.71 | **0.68*** |
| EDI-2: Social insecurity | 6.67 | 4.70 | 8.13 | 5.72 | 3.30 | .085 | .012 | 1.45 | 0.28 |
| EDI-2: Total score | 80.52 | 42.94 | 107.86 | 42.99 | 20.24 | **<.001** | .069 | 27.34 | **0.64*** |
| SCL-90R: Somatization | 1.49 | 1.00 | 1.93 | 0.90 | 10.42 | **.002** | .037 | 0.44 | 0.46 |
| SCL-90R: Obsessive/comp. | 1.51 | 0.85 | 1.99 | 0.81 | 16.58 | **<.001** | .058 | 0.49 | **0.59*** |
| SCL-90R: Interpersonal sen. | 1.68 | 0.91 | 2.18 | 0.92 | 14.27 | **<.001** | .050 | 0.50 | **0.55*** |
| SCL-90R: Depressive | 1.84 | 0.95 | 2.34 | 0.90 | 13.90 | **<.001** | .049 | 0.50 | **0.54*** |
| SCL-90R: Anxiety | 1.22 | 0.79 | 1.74 | 0.87 | 16.91 | **<.001** | .059 | 0.52 | **0.62*** |
| SCL-90R: Hostility | 1.09 | 0.87 | 1.46 | 0.98 | 7.01 | **.009** | .025 | 0.37 | 0.40 |
| SCL-90R: Phobic anxiety | 0.77 | 0.73 | 1.09 | 0.90 | 6.40 | **.012** | .023 | 0.32 | 0.39 |
| SCL-90R: Paranoid Ideation | 1.10 | 0.69 | 1.57 | 0.81 | 18.38 | **<.001** | .064 | 0.48 | **0.63*** |
| SCL-90R: Psychotic | 1.01 | 0.67 | 1.42 | 0.79 | 13.42 | **<.001** | .047 | 0.41 | **0.56*** |
| SCL-90R: GSI score | 1.38 | 0.72 | 1.85 | 0.72 | 19.83 | **<.001** | .068 | 0.47 | **0.65*** |
| SCL-90R: PST score | 57.11 | 22.89 | 66.28 | 15.34 | 12.79 | **.001** | .045 | 9.16 | 0.47 |
| SCL-90R: PSDI score | 2.04 | 0.55 | 2.42 | 0.58 | 21.08 | **<.001** | .072 | 0.38 | **0.67*** |

FA: food addiction diagnosis. ED: eating disorder. MD: mean difference. *eta^2^*: Partial eta^2^.

*^1^p:* includes Bonferroni-Finner correction for multiple statistical comparisons.

Bold: significant comparison (.05 level). *Bold: moderate (|*d*|>0.50) to high ((|*d*|>0.80) effect size.

**Table S2**. Differences on behavioral addictions for patients with negative versus positive screening for food addiction: logistic regression adjusted by age and ED subtype.

|  | FA=negative | FA=positive | Logistic adjusted by patients’ age and ED subtype | | | | | |
| --- | --- | --- | --- | --- | --- | --- | --- | --- |
|  | *n*=70 | *n*=208 | Wald_df=1_ | *^1^p* | OR | 95% CI | | \|*d\|* |
| Gambling behavior | 0.0% | 3.0% | --- | --- | --- | --- | --- | --- |
| Kleptomania behavior | 4.8% | 7.5% | 0.60 | .500 | 1.69 | 0.45 | 6.42 | 0.60 |
| Stealing behavior | 20.6% | 39.2% | 7.66 | **.033** | 2.76 | 1.34 | 5.67 | 7.66 |
| Compulsive buying behavior | 6.2% | 14.3% | 2.84 | .175 | 2.63 | 0.85 | 8.07 | 2.84 |
| Alcohol use | 33.1% | 29.7% | 0.24 | .623 | 0.86 | 0.46 | 1.59 | 0.24 |
| Tobacco use | 3.9% | 12.1% | 3.53 | .170 | 4.25 | 0.94 | 19.26 | 3.53 |
| Other drugs use | 8.3% | 14.5% | 1.73 | .269 | 2.01 | 0.71 | 5.66 | 1.73 |

FA: food addiction screening. ED: eating disorder. MD: mean difference. --- Not estimable due to the extreme low prevalence.

*^1^p:* includes Bonferroni-Finner correction for multiple statistical comparisons.

Bold: significant comparison (.05 level). *Bold: moderate (|*d*|>0.50) to high ((|*d*|>0.80) effect size.

**Table S3**. Comparison between diagnostic subtypes for the subscales of the Yale Food Addiction Scale.

|  | Total  *n*=278 | | AN  *n*=68 | | BN  *n*=110 | | OSFED  *n*=61 | | BED  *n*=39 | | *χ^2^* | df | *p* |
| --- | --- | --- | --- | --- | --- | --- | --- | --- | --- | --- | --- | --- | --- |
| Consumed more than planned | 54.3% | | 22.1% | | 79.1% | | 31.1% | | 76.9% | | 76.95 | 3 | <.001 |
| Unable to cut down or stop | 95.7% | | 89.7% | | 99.1% | | 93.4% | | 100.0% | | 11.48 | 3 | .009 |
| Great deal of time spent | 68.3% | | 42.6% | | 83.6% | | 59.0% | | 84.6% | | 39.87 | 3 | <.001 |
| Important activities given up | 72.7% | | 60.3% | | 86.4% | | 57.4% | | 79.5% | | 23.72 | 3 | <.001 |
| Use despite consequences | 56.8% | | 54.4% | | 63.6% | | 31.1% | | 82.1% | | 28.75 | 3 | <.001 |
| Tolerance | 65.1% | | 45.6% | | 79.1% | | 45.9% | | 89.7% | | 41.20 | 3 | <.001 |
| Withdrawal | 63.3% | | 36.8% | | 78.2% | | 55.7% | | 79.5% | | 37.00 | 3 | <.001 |
| Impairment or distress | 82.0% | | 73.5% | | 91.8% | | 70.5% | | 87.2% | | 16.68 | 3 | .001 |
| Food addiction: screening positive | 74.8% | | 55.9% | | 89.1% | | 62.3% | | 87.2% | | 33.08 | 3 | <.001 |
|  | Mean | SD | Mean | SD | Mean | SD | Mean | SD | Mean | SD | *F* | df | *p* |
| Food addiction: total criteria | 4.76 | 1.89 | 3.51 | 1.71 | 5.69 | 1.46 | 3.74 | 1.72 | 5.92 | 1.36 | 42.93 | 3; 274 | <.001 |

AN: anorexia. BED: binge eating disorder. BN: bulimia. ED: eating disorder. OSFED: Other Specified Feeding or Eating Disorders. SD: standard deviation.


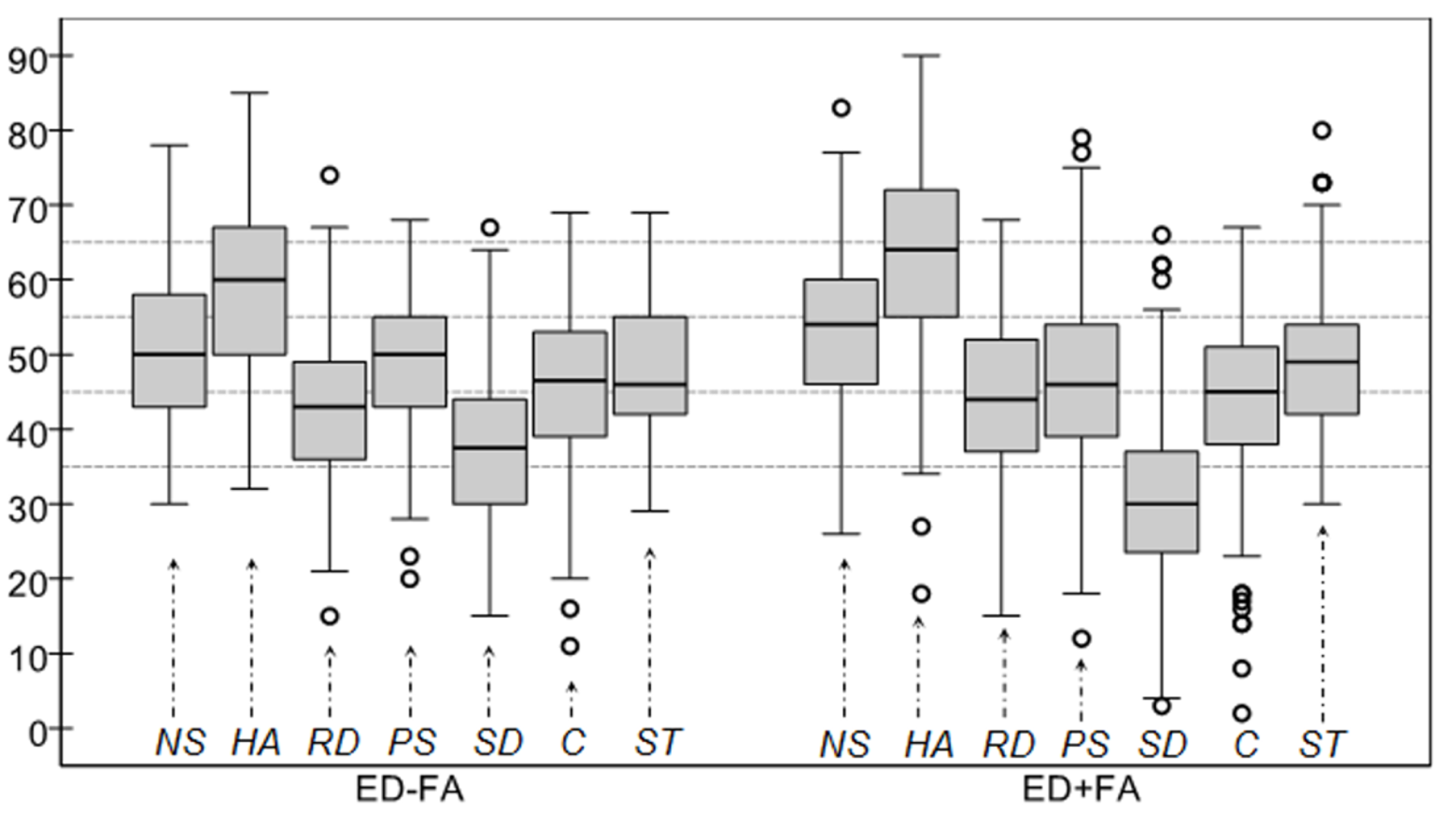


**Figure S1. Box-plot of TCI-R T-scores.** This figure shows the clinical interpretation of temperament and character traits for eating disorder patients with (ED+FA) and without (ED-FA) food addiction. Dashed lines indicate norm distributions: values between 55 and 65 or 35 and 45 are considered as subclinical, values higher than 65 or lower than 35 are considered as clinical. Comparisons are adjusted for age and ED subtype. Means in Self-Directedness were significantly different between groups, most patients with food addiction having clinically low values.

ED-FA = eating disorder patients without food addiction; ED+FA = eating disorder patients with food addiction; TCI-R: Temperament and Character Inventory- Revised; NS = Novelty Seeking; HA = Harm Avoidance; RD = Reward Dependence; P = Perseverance; SD = Self-Directedness; C = Cooperativeness; ST = Self-Transcendence
